# Coherent Phonon‐Induced Gigahertz Optical Birefringence and Its Manipulation in SrTiO_3_

**DOI:** 10.1002/advs.202205707

**Published:** 2023-01-16

**Authors:** Tao Sun, Chun Zhou, Hongli Guo, Zhi Meng, Xinyu Liu, Zhou Wang, Han Zhou, Yuming Fei, Kang Qiu, Fapei Zhang, Bolin Li, Xuetao Zhu, Fang Yang, Jimin Zhao, Jiandong Guo, Jin Zhao, Zhigao Sheng

**Affiliations:** ^1^ Anhui Key Laboratory of Condensed Matter Physics at Extreme Conditions High Magnetic Field Laboratory HFIPS Anhui, Chinese Academy of Sciences Hefei 230031 P. R. China; ^2^ University of Science and Technology of China Hefei 230026 P. R. China; ^3^ ICQD/Hefei National Laboratory for Physical Sciences at Microscale and CAS Key Laboratory of Strongly‐Coupled Quantum Matter Physics and Department of Physics University of Science and Technology of China Hefei 230026 P. R. China; ^4^ Beijing National Laboratory for Condensed Matter Physics Institute of Physics Chinese Academy of Sciences Beijing 100190 P. R. China; ^5^ Present address: Institute of Plasma Physics HFIPS Chinese Academy of Sciences Hefei 230031 P. R. China

**Keywords:** coherent phonon, gigahertz, optical birefringence, optical manipulation, ultrafast optics

## Abstract

Birefringence, which modulates the polarization of electromagnetic wave, has been commercially developed and widely used in modern photonics. Fostered by high‐frequency signal processing and communications, feasible birefringence technologies operating in gigahertz (GHz) range are highly desired. Here, a coherent phonon‐induced GHz optical birefringence and its manipulation in SrTiO_3_ (STO) crystals are demonsrated. With ultrafast laser pumping, the coherent acoustic phonons with low damping are created in the transducer/STO structures. A series of transducer layers are examined and the optimized one with relatively high photon–phonon conversion efficiency, i.e., semiconducting LaRhO_3_ film, is obtained. The most intriguing finding here is that, by virtue of high sensitivity to strain perturbation of STO, GHz optical birefringence can be induced by the coherent acoustic phonons and the birefringent amplitudes possess crystal orientation dependence. Optical manipulation of both coherent phonons and its induced GHz birefringence by double pump technique are also realized. These findings reveal an alternative mechanism of ultrafast optical birefringence control, and offer prospects for applications in high‐frequency acoustic‐optics devices.

## Graphical abstract

To address the challenge of high‐frequency signal processing, a gigahertz (GHz) optical birefringence has been developed here. With ultrafast laser pumping, the coherent acoustic phonons with low damping are created in the transducer/SrTiO_3_. By virtue of high sensitivity to strain, GHz optical birefringence is induced subsequently in SrTiO_3_ and it can also be effectively modulated by optical pumping.

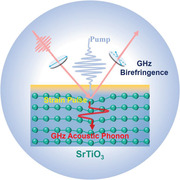

## 1 Introduction

Shaping the light, via specifically optical properties of a matter, is the main topic in modern photonics.^[^ ^1^ ^]^ Birefringence is one of the key specifics, which arises from the electrically anisotropic of a material and it can modulate the polarization vector of an electromagnetic wave deterministically.^[^ ^2^ ^]^ Favored by the wide range of applications, devices using birefringence have been commercially developed with long history, such as acousto‐optic modulators/shifters,^[^ ^3^, ^4^, ^5^ ^]^ dispersive filters,^[^ ^6^ ^]^ polarization converters,^[^ ^7^ ^]^ and retarder plates. Most of these devices can only work well up to megahertz (MHz) frequency.^[^ ^8^ ^]^ Recently, as required from the next‐generation of signal processing and communication, feasible technologies with efficient ultrafast mechanisms in the GHz range are highly desired.

Quantum optomechanics, which reversibly interconnects light with mechanical oscillators at the level of individual quanta, can provide a technical route to transfer information and energy at high‐frequency between different physical systems.^[^ ^9^ ^]^ In particular, by using an ultrafast laser technique, coherent phonons with GHz even THz frequency can be produced.^[^ ^10^, ^11^, ^12^, ^13^ ^]^ Aiming to the practical application, further efforts are being made especially for the improvement of photon–phonon conversion efficiency and the discovery of derivative effects of ultrafast acoustic phonons.^[^ ^14^, ^15^, ^16^ ^]^ As a high‐frequency strain wave, the photon produced coherent acoustic phonons accompanied by the coherent vibration of both electrons and lattice, which would provide a possible solution for ultrafast light processing,^[^ ^17^, ^18^ ^]^ such as ultrafast birefringence.

It has been well established that mechanical deformation can drive intrinsically isotropic materials to show birefringent properties when anisotropy is induced.^[^ ^19^, ^20^ ^]^ Hence, it can be expected that ultrafast birefringence might be realized in an isotropic material by coherent acoustic phonons, which would meet the requirement for high‐speed light shaping in next‐generation photonics. This is thereby an urgent need, but it is still a significant challenge before the two basic requirements are satisfied. First is the creation of strong enough and fast enough coherent acoustic phonons. The second one is the appropriate material, which should be sensitive enough to the lattice perturbation caused by the coherent phonons. Here, we demonstrate a coherent phonon‐induced GHz optical birefringence and its manipulation in the SrTiO_3_(STO) crystals. By utilizing ultrafast laser pumping and transducer optimization, the strong enough coherent acoustic phonons with GHz frequency and a low damping constant are created in the LaRhO_3_(LRO)/STO structure. It is interesting to find that, enabling power of high sensitivity to strain perturbation in STO crystal, the created ultrafast coherent acoustic phonons can produce transient birefringent with GHz frequency in STO crystal and such phonons induced GHz optical birefringence is dependent on STO crystal orientation. Furthermore, the optical manipulation of both the coherent phonons and its induced optical birefringence is realized via double pump technique.

## 2 Results and Discussion

### 2.1 Coherent Phonons in LRO/STO Structure

**Figure 1:**
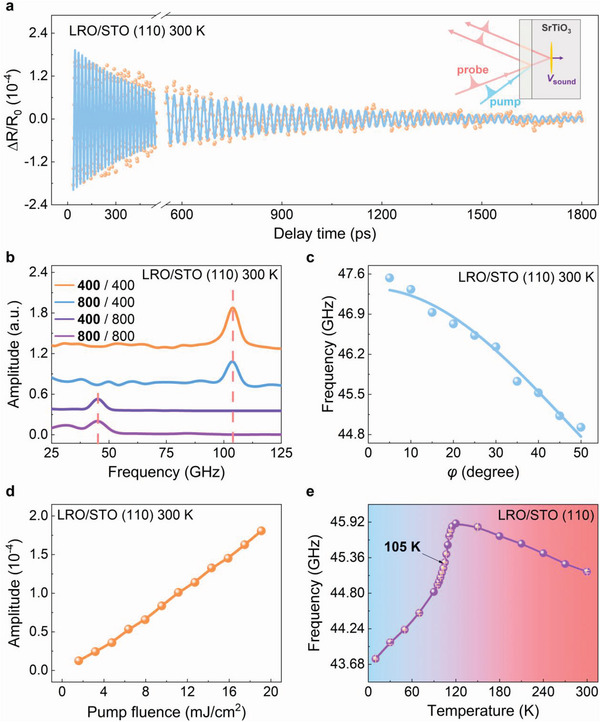
Experimental scheme and transient reflectivity data of the LRO/STO (110) structure. a) The transient reflectivity oscillation spectra (∆R) of the LRO(68 nm)/STO (110) structure measured at 300 K. The solid curves show the fitted data. The inset shows the principles of opto‐acoustic‐optic depth profiled by ultrafast acoustic interferometry (time‐domain Brillouin scattering). b) Fast Fourier transform (FFT) spectra of the transient reflectivity oscillation of LRO/STO (110) structure at different pump and probe wavelengths. The bold fonts in the legend represent the pump light wavelength. c) The frequency of the transient reflectivity oscillation versus the incident angle for the probe beam. The solid curves show the fitted data. d) The amplitude of the transient reflectivity oscillation as a function of the pump fluence. e) The transient reflectivity oscillation frequency of LRO/STO (110) structure as a function of temperature.

The time‐resolved all‐optical pump‐probe technique is utilized for the creation and detection of acoustic phonons in transducer/STO structures. The schematic of the experimental principles is shown in the inset of **Figure** **1**a. When an ultrashort optical pump pulse is partially absorbed by a transducer layer, a strain pulse is generated by instantaneous thermal expansion,^[^ ^21^ ^]^ which could be viewed as a wave packet arising from the superposition of longitudinal acoustic phonons having different wave vectors.^[^ ^22^ ^]^ The acoustic pulse travels from the transducer layer into the STO layer and induces a refractive index modulation, which constitutes a traveling scattering plane. The delayed optical probe pulse is reflected by the scattering planes through the stimulated Brillouin scattering process.^[^ ^23^ ^]^ The interference between probe beams reflected by the interface and reflected by the propagating strain scattering planes are responsible for the transient oscillations in the measurement of reflectivity changes ΔR/R_0_. The typical results of the LRO/STO (110) structure measured at 300 K are shown in Figure 1a. The oscillation is obtained by subtracting the exponential‐like decay background and is ascribed to the longitudinal coherent acoustic phonons. The amplitude of the ∆R/R_0_ oscillation (*A*_osc_) is up to ≈2 × 10^−4^. Interestingly, such oscillation holds a long damping tail with the order of a few nanoseconds. The fitting results with a damped cosine function show that the oscillatory frequency (*f*) is ≈45.3 GHz and the damping constant is as low as ≈0.007. Such low damping property indicates that the STO crystal is a good transmission carrier for the coherent acoustic phonons.

There are several ways to produce coherent phonons, including strain pulse,^[^ ^10^, ^21^ ^]^ impulsive stimulated Raman scattering,^[^ ^24^ ^]^ and displacive excitation of coherent phonons.^[^ ^25^ ^]^ In the case of the strain pulse model, the *f* depends on the probe wavelength (*λ*_probe_) and the incident angle (*φ*_probe_) but is independent of the pump wavelength (*λ*_pump_). Contrarily, the *f* depends on *λ*_pump_ in the other two models.^[^ ^24^, ^25^ ^]^ Figure 1b shows the fast Fourier transform (FFT) of the oscillations from the ∆R/R_0_ traces of LRO/STO (110) structure at different *λ*_pump_ and *λ*_probe_ (Figure S1, Supporting Information). It is found that the *f* increases from ≈45.3 to ≈104.1 GHz with *λ*_probe_ changing from 800 to 400 nm and it is independent on the *λ*_pump_. Moreover, for strain pulse‐induced coherent phonons, the *f* is dependent on the *φ*_probe_ as^[^ ^21^ ^]^

(1) $$f=\frac{2v_{s}\sqrt{n^{2}-\sin^{2}\varphi_{\mathrm{probe}}}}{\lambda_{\mathrm{probe}}}$$

where *v*_s_ is the sound velocity, the *n* is the refractive index of the material. The *f* – *φ*_probe_ relationship (Figure S2, Supporting Information) can be well fitted with Equation (1) as shown in Figure 1c (solid line). These two features certainly identified the observed coherent phonons as strain pulse waves, which provides the basis for the subsequently derived phenomena.

With the refractive index *n* of STO ≈2.3422 at 800 nm,^[^ ^26^ ^]^ the *v*_s_ of the strain wave in STO can be obtained from the fitting results as ≈8088 m s^−1^.^[^ ^27^ ^]^ Further experiments with different LRO transducer layer thicknesses have been conducted, and the results show that the transducer layer thickness does not change the frequency but affects the starting time and the magnitude of the transient reflectivity oscillations (Figure S3, Supporting Information). In detail, the starting times are ≈12, ≈22, and ≈34 ps for the LRO/STO structure with ≈17, ≈68, and ≈136 nm thick LRO layer, respectively. The starting time of the oscillation is the time that the strain pulse propagates from the surface of the LRO film to the LRO/STO interface at the speed of the sound in LRO (inset of Figure 1a). On the one hand, according to the thickness dependence of the starting time, the sound speed in LRO film is estimated as ≈4900 m s^−1^. On the other hand, the absence of oscillation before the starting time indicates that the observed oscillations are only related to the propagation of coherent acoustic phonons in the STO crystal, which can help us extract acoustic information of STO itself from the transducer/STO structure.

After the creation, detection, and identification, the pumping fluence and temperature dependence of the coherent acoustic phonons have also been explored. As shown in Figure 1d, the *A*_osc_ is almost linearly proportional to the pumping fluence. And it seems that there is no pumping fluence threshold for the creation of the coherent acoustic phonons in STO, which implies the high energy transmission efficiency of the strain wave between LRO and STO (Figure S4, Supporting Information). Figure 1e presents the temperature dependence of *f*, which increases slowly with decreasing temperature, and then gets a sharp drop ≈105 K (Figure S5, Supporting Information). This indicates that the STO crystal becomes soft and the velocity of longitudinal acoustic phonons drops at the antiferrodistortive phase transition temperature *≈*105 K due to the superelastic effect.^[^ ^28^ ^]^

### 2.2 Transducer Layer Effect on the Coherent Phonons

**Figure 2:**
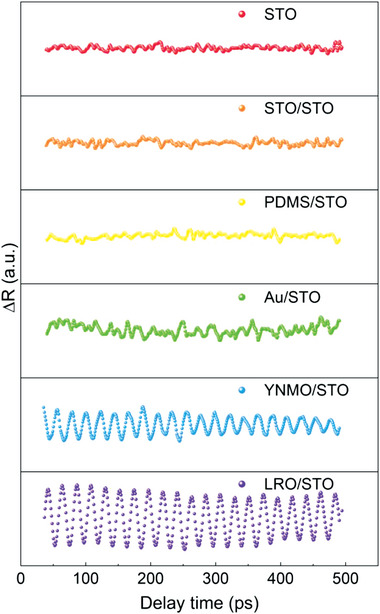
Reflectivity changes as a function of delay time for different transducer layers on the STO (110) substrate at 300 K. STO: STO substrate; STO/STO: an STO thin film on the STO substrate; PDMS/STO: a polydimethylsiloxane film on the STO substrate; Au/STO: a gold film on the STO substrate; YNMO/STO: an Y_2_NiMnO_6_ film on the STO substrate; LRO‐STO: a crystallized LaRhO_3_ film on the STO substrate.

The creation of coherent acoustic phonons in transducer/STO structure is an optomechanical process in which the quantum state transitions between different physical systems are accompanied by photon–phonon energy conversion. The above data have demonstrated that the STO crystal is a good carrier for the coherent phonons, while the coherent phonons creation, i.e., the photon–phonon conversion process, relies heavily on the transducer layers and the interfaces.^[^ ^14^, ^15^, ^29^ ^]^ As shown in **Figure** **2**, no coherent phonons can be detected in the bare STO and STO film (≈39 nm)/STO samples, indicating the disablement of the optomechanical effect at the air/STO interface and the requirement of a suitable transducer for the STO crystal. Previously, polydimethylsiloxane (PDMS) film and thin metallic film have been used as optoacoustic transducers for different transmitter materials.^[^ ^30^, ^31^, ^32^ ^]^ In this work, both PDMS film and gold film (≈34 nm) are also tested as transducers on the STO crystal. It is found that negligible and weak coherent phonons oscillation can be observed in PDMS/STO and Au/STO structures, respectively. The low absorbance in PDMS and high reflectivity in Au might be the main reasons for the poor optoacoustic conversion efficiency in both PDMS/STO and Au/STO structures.^[^ ^33^ ^]^

In general, the transducer layer should possess small thermal conductivity, low specific heat capacity, as well as high absorption coefficient.^[^ ^14^ ^]^ In order to improve the photon–phonon conversion efficiency further, two perovskite oxides with a similar structure to the STO and meeting the key characteristics of photoacoustic transducer applications have become the candidate objects of our attention.^[^ ^34^, ^35^, ^36^, ^37^ ^]^ We deposited dielectric Y_2_NiMnO_6_ (YNMO) film and semiconducting LRO film on the STO crystals, respectively, by the pulsed laser deposition technique.^[^ ^38^, ^39^ ^]^ As shown in Figure 2, there are obvious transient reflectivity oscillation, as well as coherent acoustic phonons, can be observed in both YNMO/STO and LRO/STO structures. The *f* of the coherent phonons in these two structures are the same (Figure S6b, Supporting Information). While, the oscillation amplitude *A*_osc_ in LRO/STO structure is larger than that in YNMO/STO case, suggesting a higher photon–phonon energy conversion. Furthermore, it is interesting to find that the *A*_osc_ of the crystallized LRO sample is larger than that of the amorphous LRO case (Figure S6a, Supporting Information). These results illustrate that, besides the photon absorbance, the acoustic impedance matching between the transducer and the STO crystal also plays vital roles in the photon–phonon conversion process. In short, better transducer layer and acoustic impedance matching, better the photon–phonon conversion efficiency.

### 2.3 Coherent Phonons‐Induced Optical Birefringence in STO

**Figure 3:**
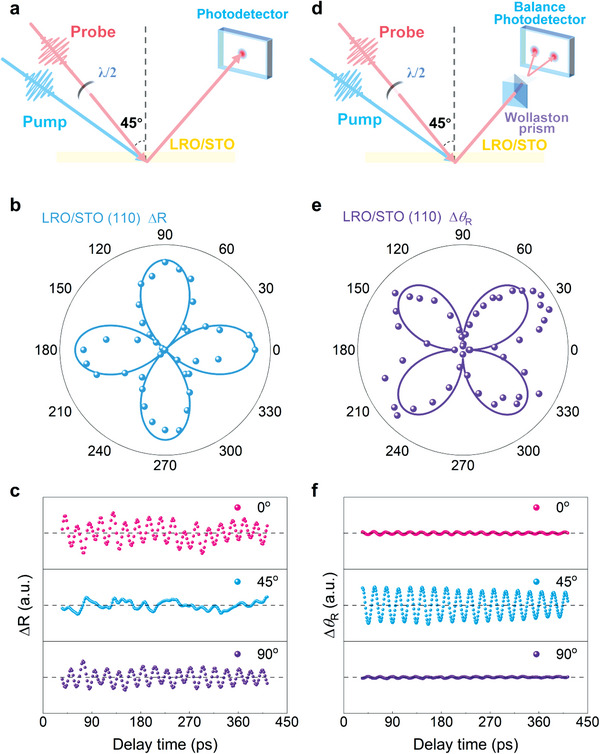
The experimental setup and polarization‐dependent data for the LRO/STO (110) heterostructure. a) The experimental optical setup for the transient reflectivity oscillation spectra. b) The oscillatory amplitude of TR‐ΔR versus the polarization angle of the input probe beam. c) Transient reflectivity oscillation spectra at different polarization angles of 0° (**p**‐polarization), 45°, and 90° (**s**‐polarization). d) The experimental optical setup for the optical birefringence oscillation spectra. e) The oscillatory amplitude of the optical birefringence (TR‐Δ*θ*_R_) versus polarization angle of the input probe beam. f) Time‐resolved optical birefringence (TR‐Δ*θ*_R_) at different polarization angles of the input probe beam (0°, 45°, and 90°).

As mentioned above, the transient reflectivity oscillation (TR‐ΔR) is the interference between probe laser beams reflected from the LRO/STO interface and from the traveling scattering plane in STO, respectively. The polarization dependence of *A*_osc_ depends on the phase difference *δ*_s_‐*δ*_p_, where *δ*_s_ (*δ*_p_) are the interferential phase difference between **s** (**p**) polarized probe beams reflected from the LRO/STO interface and from the traveling scattering plane. We numerically simulated the amplitude and phase of TR‐ΔR with the variation of the polarization angle of the probe beam (Figure S7, Supporting Information). It is found that the polarization dependence of *A*_osc_ has fourfold symmetry, and the maximum amplitude occurs when the probe beam polarization is set as 0°, 90°, 180°, and 270°. Our experimental observations are consistent with the simulation results as shown in **Figure** **3**b,c. Additionally, the phase difference *δ*_s_‐*δ*_p_ are 0, 180, and 0 degrees for LRO/STO (100), LRO/STO (110), and LRO/STO (111) structures, respectively (Figure S8, Supporting Information). It indicates that the laser‐induced strain wave in STO crystal is anisotropic, which will be discussed further below.

At room temperature, the STO crystal is cubic and optically isotropic without birefringence.^[^ ^40^, ^41^ ^]^ With a static strain applied, the anisotropic lattice deformation can drive the STO from centrosymmetric to non‐centrosymmetric and then bring a static birefringence.^[^ ^40^, ^41^, ^42^ ^]^ Here, the coherent phonons are created by ultrafast laser pumping, and such strain wave could induce transient lattice deformation as well as its birefringence.^[^ ^18^ ^]^ To explore this issue, the time‐resolved polarization rotation angle measurement (TR‐Δ*θ*_R_) is employed as schematically shown in Figure 3d. Different from the TR‐ΔR, the TR‐Δ*θ*_R_ can reveal the optical birefringence and the refractive index anisotropy of the crystal efficiently.^[^ ^18^, ^43^ ^]^ It is interesting to find that distinct TR‐Δ*θ*_R_ signals, i.e., optical birefringence, can be detected in the laser‐pumped LRO/STO (110) structure (Figure S9, Supporting Information). The periodic oscillation of TR‐Δ*θ*_R_ has the same frequency as TR‐ΔR ≈45 GHz. Moreover, the amplitude of the induced birefringence has no obvious attenuation within 400 ps and there is still a slight oscillation even at 2000 ps with a low damping constant of ≈0.007. The polarization dependences of the coherent phonons‐induced birefringence are summarized in Figure 3e,f. The polar pattern of the Δ*θ*_R_ amplitude shows a fourfold symmetry. Different from the TR‐ΔR results, the phonons induce birefringence (TR‐Δ*θ*_R_) reaches the maximum amplitude at 45^o^, 135^o^, 225^o^, and 315^o^, in agreement with our numerically simulated results (Figure S10, Supporting Information). These results indisputably show that the ultrafast coherent acoustic phonons can induce GHz optical birefringence in STO crystals efficiently.

The same kind of experiment has been carried out in (La,Sr)(Al,Ta)O_3_ (LAST) crystal and LRO/LAST bilayered structures (Figure S11, Supporting Information). Similar to the bare STO case, no coherent phonons can be observed in the bare LAST crystal. By adding an LRO transducer layer, the TR‐ΔR oscillation with a frequency of ≈35 GHz can be detected. The sound speed in LAST is estimated to be ≈7300 m s^−1^, which is slower than that in STO (≈8088 m s^−1^). Surprisingly, distinct from the STO case, the transient optical bireference (TR‐Δ*θ*_R_ oscillation) is negligible in the LRO/LAST structure (Figure S11a, Supporting Information). That means that, with the same kind of coherent phonons pumped by the ultafast laser, the optical refringence can be induced in STO but it is absent in LAST. Such difference indicates the refractive index ellipsoid of STO crystal is more sensitive to the strain pulse than that in LAST. Both theoretic and experimental studies have revealed that STO is an incipient ferroelectric or a quantum paraelectric, i.e., it does not undergo a ferroelectric transition even down to low temperatures.^[^ ^44^ ^]^ However, this quantum paraelectric state is very sensitive to lattice perturbations.^[^ ^45^, ^46^ ^]^ In our case, the ultrafast laser‐produced coherent acoustic phonons can act as “ultrafast strain engineering” and exert perturbations on the STO lattice. Just because of the sensitivity to the lattice perturbation, the GHz optical birefringence can be produced in STO crystal. Contrarily, for the LAST case, it is negligible due to its insensitivity. This finding suggests that the creation of phonons‐induced GHz birefringence needs not only the high‐frequency coherent phonons but also the appropriate materials which are sensitive to lattice perturbation. Accordingly, some novel materials, such as two‐dimensional van der waals materials possessing intrinsic structural anisotropy and high sensitivity to the lattice perturbation,^[^ ^47^, ^48^, ^49^ ^]^ may be good candidates for the GHz birefringence generation.

### 2.4 Anisotropic Behavior of Coherent Phonon‐Induced Optical Birefringence

**Figure 4:**
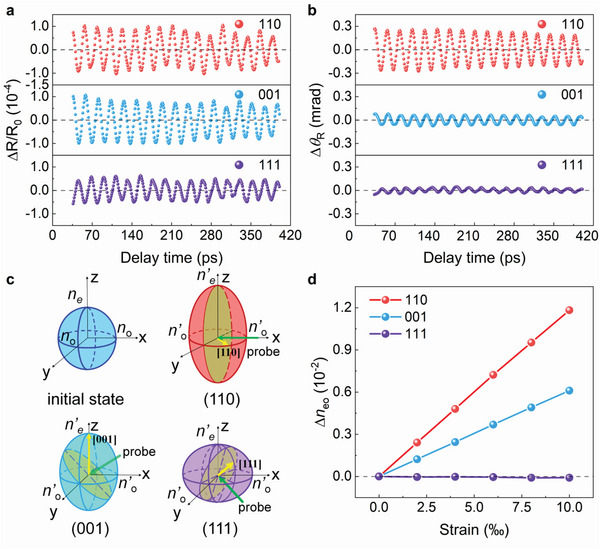
Behaviors of coherent phonon and optical birefringence with different lattice orientations of STO. a) The pure oscillatory response ΔR/R_0_ of the reflected probe beam at 0^o^ (**p**‐polarization). b) The polarization rotation angles Δ*θ*_R_ of the probe beam at 45°‐polarization. c) Schematic sketch of refractive index ellipsoid stretching of STO in different orientations (001), (110), and (111). The yellow and green arrows represent the surface normal direction of the sample and the direction of the incident probe beam, respectively. The shaded plane represents the refractive index elliptic plane perpendicular to the incident probe beam. d) First‐principles calculation of strain‐induced birefringence of STO in different orientations (001), (110), and (111).

Intrinsic optical birefringence originates from the intrinsic lattice anisotropy of the crystal.^[^ ^40^ ^]^ In this context, it is worthy to explore the crystal orientation effects on both coherent phonons and its induced birefringence. For the coherent phonons, as shown in **Figure** **4**a, the amplitudes of coherent phonons (*A*_osc_ of ∆R/R_0_) in LRO/STO (110) and LRO/STO (001) are larger than that in LRO/STO (111). It may suggest that the (110) and (001) types of LRO/STO interfaces have better acoustic impedance matching compared with the (111) case (Figure S12, Supporting Information). In addition to the oscillation amplitude, the oscillation frequency is also orientation dependent with a tendency of *f*_(111)_ > *f*_(110)_ > *f*_(001)_. Consequently, we can deduce the relationship between sound velocity and STO crystal orientation, which is *v*_(111)_ > *v*_(110)_ > *v*_(001)_ and consistent with that reported previously.^[^ ^27^ ^]^

For the phonons‐induced optical birefringence (TR‐Δ*θ*_R_), the situation of crystal orientation dependence is much more complicated. As shown in Figure 4b, the oscillation amplitude of Δ*θ*_R_ is only 0.02 mrad for LRO/STO (111), which increases up to 0.08 mrad for LRO/STO (001). When the crystal orientation is shifted to (110), the oscillation amplitude of Δ*θ*_R_ is further enhanced up to 0.28 mrad. In order to further describe the crystal orientation dependence of phonons‐induced optical birefringence, a quantity conversion efficiency *r =* Δ*θ*_R_/[∆R/R_0_] is defined. The larger *r* means higher conversion efficiency from coherent phonon to optical birefringence. According to the data shown in Figure 4a,b, the *r* is calculated as ≈0.287, ≈0.086, and ≈0.051 for LRO/STO (110), LRO/STO (001), and LRO/STO (111), respectively. This clearly indicates that the anisotropic feature of coherent phonons‐induced optical birefringence and (110) orientation holds relatively high conversion efficiency.

For the pristine STO crystal, the refractive indices are isotropic. After laser pumping and the produced coherent acoustic phonons (strain wave) modulates the refractive indices of STO and such modulation is anisotropic as shown in Figure 4c. To understand the observed orientation‐dependent transient optical birefringence in STO, we performed density functional theory (DFT) calculations on the strain‐induced deviation of refractive index anisotropy (∆*n*_eo_ = ∆*n*_e_ − ∆*n*_o_) in STO as shown in Figure 4d. Assuming the laser‐induced strain will cause lattice deformation along the surface normal direction, the non‐zero strain was only applied to the direction perpendicular to the surface plane. In this way, the propagating acoustic wave is purely longitudinal, we can obtain the deviation of the permittivity tensor due to the acoustic strain waves, and then the ∆*n*_eo_ is derived, which corresponds to the optical birefringence of STO. The calculated result in Figure 4d agrees with the experimentally observed optical birefringence in Figure 4b, in which the magnitude of ∆*n*_eo_ in three orientations is ranked as (110) > (001) > (111). Furthermore, the ∆*n*_eo_ in (111) is negative and agrees with the observed phase reversion in (111) compared to the other two orientations shown in Figure 4b.

### 2.5 Manipulation of Coherent Phonons and Optical Birefringence

**Figure 5:**
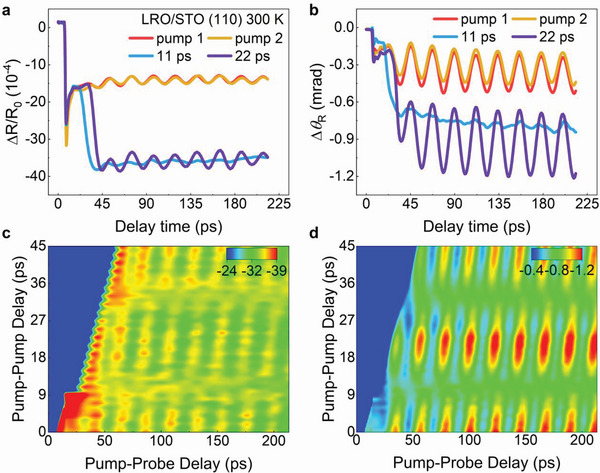
Optical manipulation of coherent phonons and optical birefringence in LRO/STO (110) heterostructure. a,b) Transient reflectivity and birefringence changes in four cases: only with pump 1 (pump 1), only with pump 2 (pump 2), with pumps 1 and 2 pulses, and the interval time of 11 ps (coherent cancellation), with pumps 1 and 2 pulses and the interval time of 22 ps (coherent enhancement). c,d) Two‐dimension image maps of the transient reflectivity and birefringence changes versus pump‐probe delay and pump–pump delay.

After the creation and detection of the coherent phonons and its induced optical birefringence in the STO crystal, the next expectation is to control them, which would promote their application in future optic‐acousto‐optic devices. Here, a coherent control method by double pump pulses was designed and performed in the LRO/STO structure. The laser pulse from a Ti:sapphire amplifier was first divided into two parts: pump and probe. The pump pulse was introduced into a home‐made Michelson‐type interferometer to produce two pump pulses (pump 1 and pump 2), and the delay between pump 1 and pump 2 (pump‐pump delay) was adjusted by a motorized stage (the detailed description can be found in Experimental Section and Figure S13, Supporting Information). The probe pulse was traveling through another delay stage to introduce the pump‐probe delay. For each pump 1 and pump 2, the acoustic coherent phonons and the optical birefringence can be created in LRO/STO (110) at 300 K, respectively (**Figure** **5**a,b). When both pump 1 and pump 2 are introduced together onto the sample surface, the amplitude of both the coherent phonons and phonons‐induced optical birefringence can be controlled by tuning the pump–pump delay. As typically shown in Figure 5a,b, both the coherent phonons and optical birefringence can be coherently enhanced nearly twice with a pump‐pump delay of 22 ps. However, they can be suppressed almost down to zero with a pump‐pump delay of 11 ps (half cycle of the acoustic phonons oscillation period). More information about the optical manipulations of both the coherent phonons and optical birefringence can be found in Figure 5c,d, in which the 2D mapping of the TR‐ΔR and TR‐Δ*θ*_R_ plotted against pump‐probe delay and pump‐pump delay are presented, respectively. One point is worth mentioning that most of the current optical birefringence devices are used in the kHz and MHz (radiofrequency) range of sound waves.^[^ ^50^, ^51^ ^]^ Here, the stimulated birefringence oscillation and its manipulation in STO are in the GHz frequency range, which could meet the high‐speed requirements of the next generation of signal processing and communication.

## 3 Conclusion

In summary, one kind of GHz optical birefringence, induced by ultrafast coherent acoustic phonons, is realized in STO crystal. In order to produce strong enough stain wave pulses, a series of transducer layers are examined and the optimized LRO/STO structure is obtained with high efficiency of photon–phonon conversion and low damping constant. Of particular interest, we discovered that the ultrafast coherent acoustic phonons can induce a GHz optical birefringence in STO due to its high sensitivity to the lattice perturbation. Moreover, by utilizing the double pump technique, both coherent phonons and induced birefringence manipulations are realized optically.

## 4 Experimental Section

### Materials

The LRO, amorphous‐LRO (A‐LRO), and YNMO thin oxide transducer films were grown on STO crystals by the pulsed laser deposition (PLD) method. The growth details were reported previously.^[^ ^34^, ^35^ ^]^ The Au thin transducer film was deposited by the thermal evaporation method. A 200 mg mL^−1^ PDMS solution in toluene was prepared utilizing the base/cross‐linker ratio of 10:1. The PDMS transducer film was then deposited by spin‐coating the PDMS solution on the STO substrate at 3000 rpm, followed by full curing at 65 °C for 1 h.^[^ ^52^ ^]^ The STO crystals are commercially available with a thickness of 0.5 mm (Hefei Kejing Materials Technology).

### Time‐Resolved Optical Reflectivity and Optical Birefringence Effect Measurement

To track the dynamics of the coherent phonons, time‐resolved optical reflectivity and optical birefringence effect measurements were performed. The samples were placed in a cryostat (Oxford, 7 T SpectromagPT), which can provide a temperature environment of 1.5–300 K. The measurement setup is based on a regenerative amplified Ti:sapphire laser (Solstice Ace, Spectra‐Physics) producing 100 fs pulses at 800 nm wavelength, and 1 kHz repetition rate. The laser pulses were divided into the pump and probe beams by a 7:3 beam splitter. By adding a nonlinear beta‐barium borate (BBO) crystal in the optical path, the wavelength of the pump and probe beams can be switched between 800 and 400 nm. The incident angle of the probe beam varied from ≈5° to 50° with respect to the normal direction of the sample plane while the pump was at 55° versus the surface normal. The beams were loosely focused onto the sample surface with a spot diameter of 200 µm by focusing lenses for the pump and probe. The incident pump fluence varied from 1.59 to 19.08 mJ cm^−2^ at a fixed probe fluence of 1.4 mJ cm^−2^. The time delay between the pump and probe pulses was controlled by a long‐range motorized linear delay stage, which can provide a maximum delay of 4 ns. The pump beam was chopped at a rate of 635 Hz to measure the relative changes in the reflectance between the pump perturbed (R_0_ + ΔR) and unperturbed (R_0_) samples. A low‐noise photodetector (New Focus, Model 2007) and a lock‐in amplifier (Zurich Instruments, MFLI 500 kHz) are used to improve the signal‐to‐noise ratio. To track the transient optical birefringence effect Δ*θ*_R_, the reflected light from the sample was first filtered to remove the pump, passed through a half‐wave plate and a Wollaston prism, and then detected by a pair of balanced photodiodes. The pump‐induced change in the rotation of the polarization angle was determined as the ratio of the intensity imbalance/the sum intensity of each photodiode obtained from a lock‐in amplifier locked at the pump modulation frequency.

### Coherent Phonons and Optical Birefringence Manipulation

To realize the coherent phonons and optical birefringence manipulation in the LRO/STO structure, time‐resolved optical reflectivity (TR‐ΔR) measurement with two pump pulses was performed. The LRO/STO (110) sample was kept at room temperature. The light source was a Ti:sapphire pulse laser with a wavelength of 800 nm, a repetition rate of 1 kHz, and a pulse duration of 100 fs. A beam splitter was used to divide the laser output into two parts. After converting the pump pulse wavelength to 400 nm with a BBO by second harmonic generation (SHG) process, the pump beam was further divided into two optical beams by another beam splitter. To control the time delay between the pump pulses of the two beams (Δ*t*), a delay stage (DS2) was placed in the one of pump beams. A positive sign of Δ*t* means that the pump pulse of the beam that passed through DS2 (the “second” pump pulse) arrived later at the sample surface than the pump pulse of the other beam (the “first” pump pulse). The relative delay (*t*) between the first pump pulse and the probe pulse is scanned by the DS1 delay stage. The incident angle of the probe beam is ≈45^o^ with respect to the normal direction of the sample plane, while the pump was ≈35° to the surface normal. The beam sizes are the same as the single pump experiment as mentioned above. Both pump 1 and pump 2 fluences are 9.6 mJ cm^−2^. The experimental configuration of the signal detection part is consistent with that of the time‐resolved light reflectivity and optical birefringence effect experiment.

### Calculation Method

The optimized atomic structures of unstrained and strained STO and the optical properties were determined using the Vienna Ab initio Simulation Package (VASP).^[^ ^53^ ^]^ The Perdew–Burke–Ernzerhof exchange‐correlation functional^[^ ^54^ ^]^ along with Projector‐Augmented Wave potentials^[^ ^55^, ^56^ ^]^ were employed in these calculations. The atomic geometry in each unit cell was fully optimized until the residual force on each atom was less than 0.01 eV Å^−1^, a 9 × 9 × 9 Monkhorst‐Pack k‐mesh was used and the energy cutoff was 400 eV. The optimized lattice constant for unstrained bulk STO is *a* = *b* = 3.914 Å and *c* = 3.907 Å. The static dielectric tensor *ε* of the unstrained bulk STO is obtained, with *ε*_xx_ = *ε*_yy_ = 6.31 and *ε*_zz_ = 6.30. The refractive index *n* is derived by $n=\sqrt{\varepsilon }$, and then the deviation of refractive anisotropy ∆(∆*n*) is further obtained.

## Conflict of Interest

The authors declare no conflict of interest.

## Author Contributions

Z.S. conceived and supervised the project. T.S. and C.Z. conducted the optical experiments. H.G. and J.Z. performed the theoretical calculation. Z.M. fabricated LRO samples. F.Y., H.Z., and M.F. provided other samples. F.Z,, J.M.Z., X.Z., F.Y., J.G., B.L., X.L., Z.W., and K.Q. gave constructive comments. Z.S., T.S., and C.Z. wrote the paper with contributions from all authors.

## Supporting information

Supporting Information

## Data Availability

The data that support the findings of this study are available in the supplementary material of this article.
