# Supplementary material for: Coherent Phonon‐Induced Gigahertz Optical Birefringence and Its Manipulation in SrTiO3
Source: Adv Sci (Weinh). 2023 Jan 16;10(7):2205707. doi: 10.1002/advs.202205707 (PMC9982545; doi:10.1002/advs.202205707)
Supplement: Supplementary file 1 — Supporting Information [file ADVS-10-2205707-s001.pdf]

Supplementary Materials for
Coherent phonon induced gigahertz optical birefringence and its manipulation in $SrTiO_3$

Tao Sun[1,2,#], Chun Zhou[1,#,†], Hongli Guo[3], Zhi Meng[1], Xinyu Liu[1], Zhou Wang[1,2], Han Zhou[1,2], Yuming Fei[1,2], Kang Qiu[1], Fapei Zhang[1], Bolin Li[1], Xuetao Zhu[4], Fang Yang[4], Jimin Zhao[4], Jiandong Guo[4], Jin Zhao[3], and Zhigao Sheng[1,]*

[1] Anhui Key Laboratory of Condensed Matter Physics at Extreme Conditions, High Magnetic Field Laboratory, HFIPS, Anhui, Chinese Academy of Sciences, Hefei 230031, P. R. China

[2] University of Science and Technology of China, Hefei 230026, P. R. China

[3] ICQD/Hefei National Laboratory for Physical Sciences at Microscale, and CAS Key Laboratory of Strongly-Coupled Quantum Matter Physics, and Department of Physics, University of Science and Technology of China, Hefei, Anhui 230026, P. R. China

[4] Beijing National Laboratory for Condensed Matter Physics, Institute of Physics, Chinese Academy of Sciences, Beijing 100190, P. R. China

[#] These authors contributed equally to this work.

[†] Present address: Institute of Plasma Physics, HFIPS, Chinese Academy of Sciences, Hefei, 230031, P. R. China.

* Corresponding authors:

Z. S. (zhigaosheng@hmfl.ac.cn).

Supplementary Figures

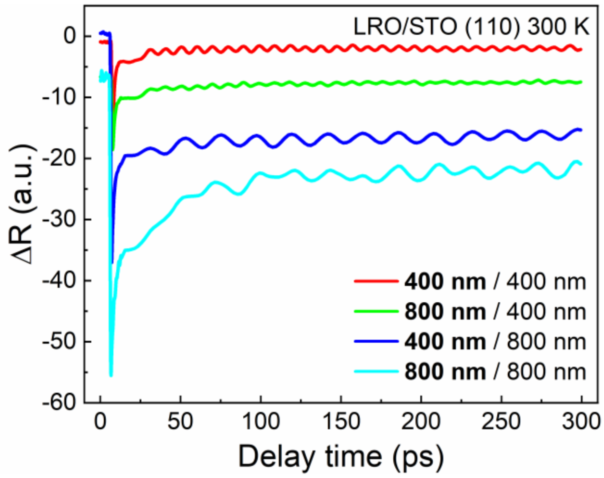

Figure S1. Transient reflectivity spectra ΔR of the LRO/STO (110) structure at different pump and probe wavelengths. The bold fonts in the legend represent the pump light wavelength.

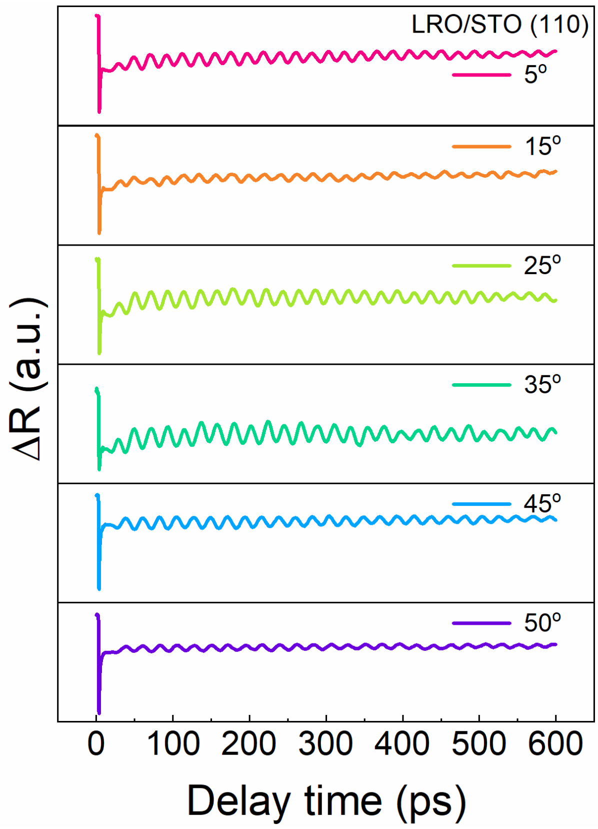

Figure S2. Transient reflectivity spectra ΔR of the LRO/STO (110) structure under different incident angles of the probe beam. The incident angles are varied from 5° to 50°.

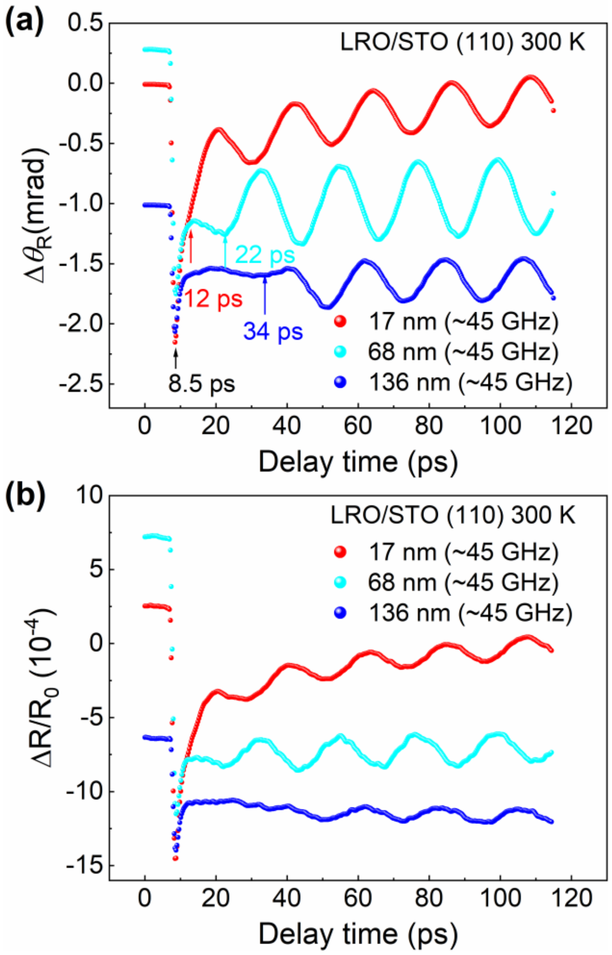

Figure S3. (a), (b) Optical birefringence $\Delta\theta_R$ and reflectivity spectra $\Delta R/R_0$ versus the delay time for three LRO films with different thicknesses on the STO (110) substrate. The thicknesses of LRO films are around 17 nm, 68 nm, and 136 nm, respectively.

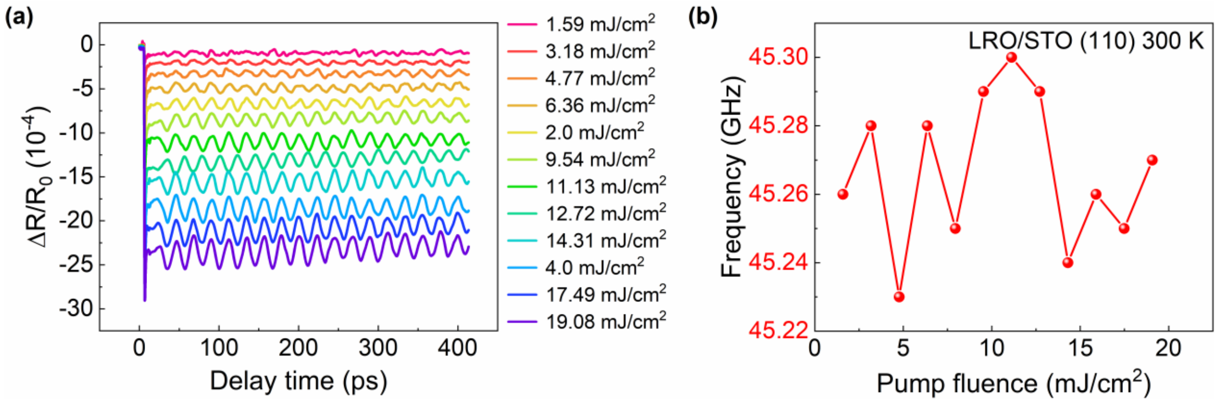

Figure S4. Transient reflectivity and frequency data of the LRO/STO (110) structure under different pump fluences. (a) Transient reflectivity spectra $\Delta R/R_0$ under different pump fluences from 1.59 mJ cm^{-2} to 19.08 mJ cm^{-2}. (b) The frequency of the experimental reflectivity oscillation as a function of the pump fluence.

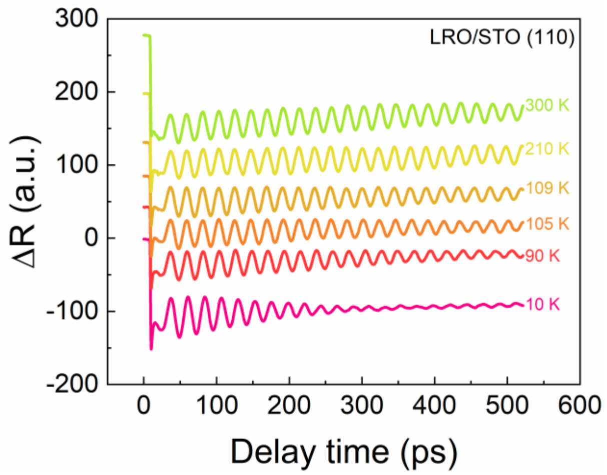

Figure S5. Temperature-dependent transient reflectivity spectra ΔR of the LRO/STO (110) structure. The temperatures are changed from 10 K to 300 K.

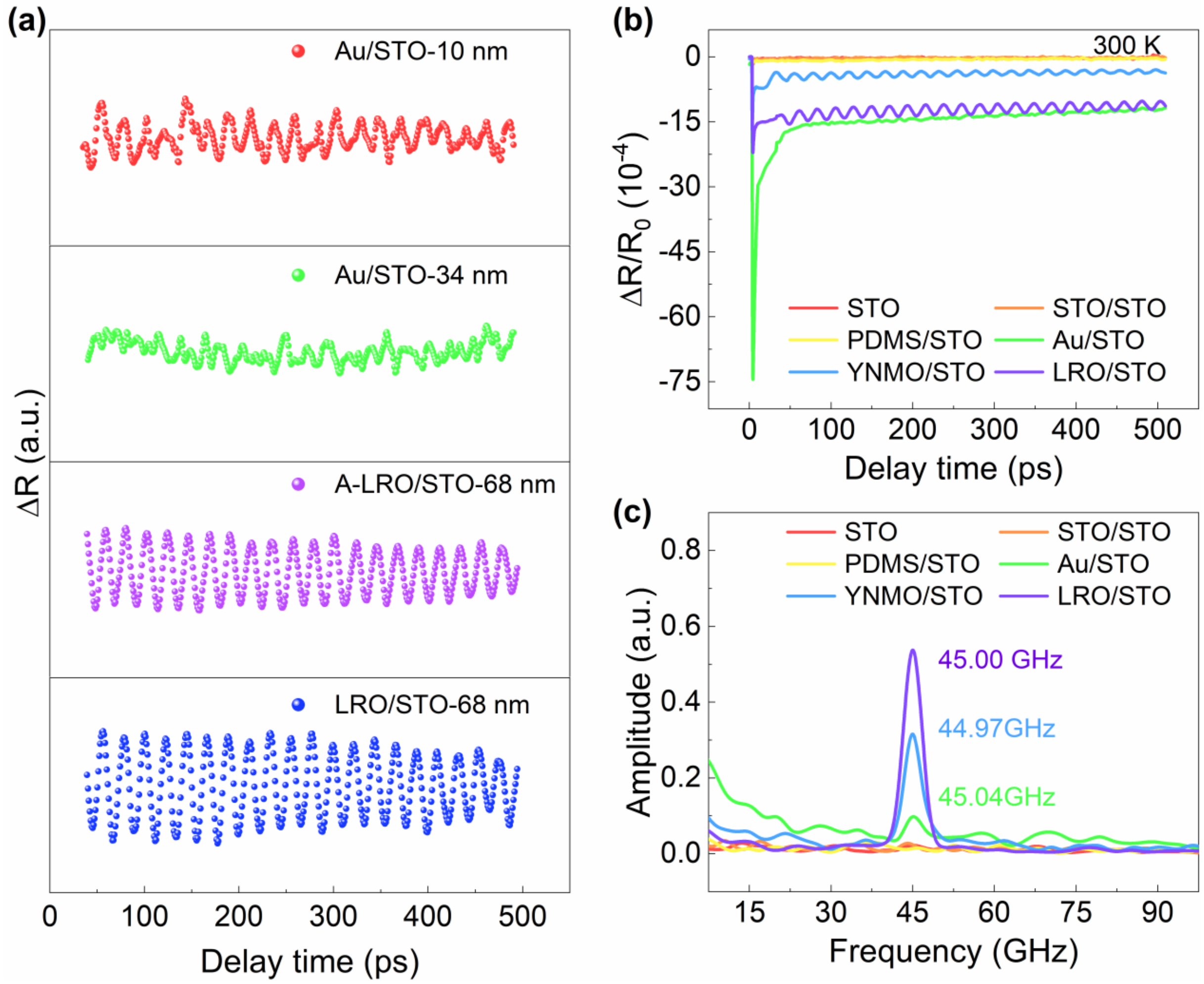

Figure S6. Transient reflectivity data for different transducer layers on STO substrate. (a) Transient reflectivity oscillation spectra for different transducer layers on the STO (110) substrate. LRO-STO: a crystallized $LaRhO_3$ film on the STO substrate; A-LRO/STO: an amorphous $LaRhO_3$ film on the STO substrate. (b) Transient reflectivity spectra $\Delta R/R_0$ for different transducer layers on STO substrate. (c) The corresponding fast Fourier transform of the oscillation signal in Figure S6(b). The central frequencies are the same for STOs with different transducer layers, which are around ~45 GHz.

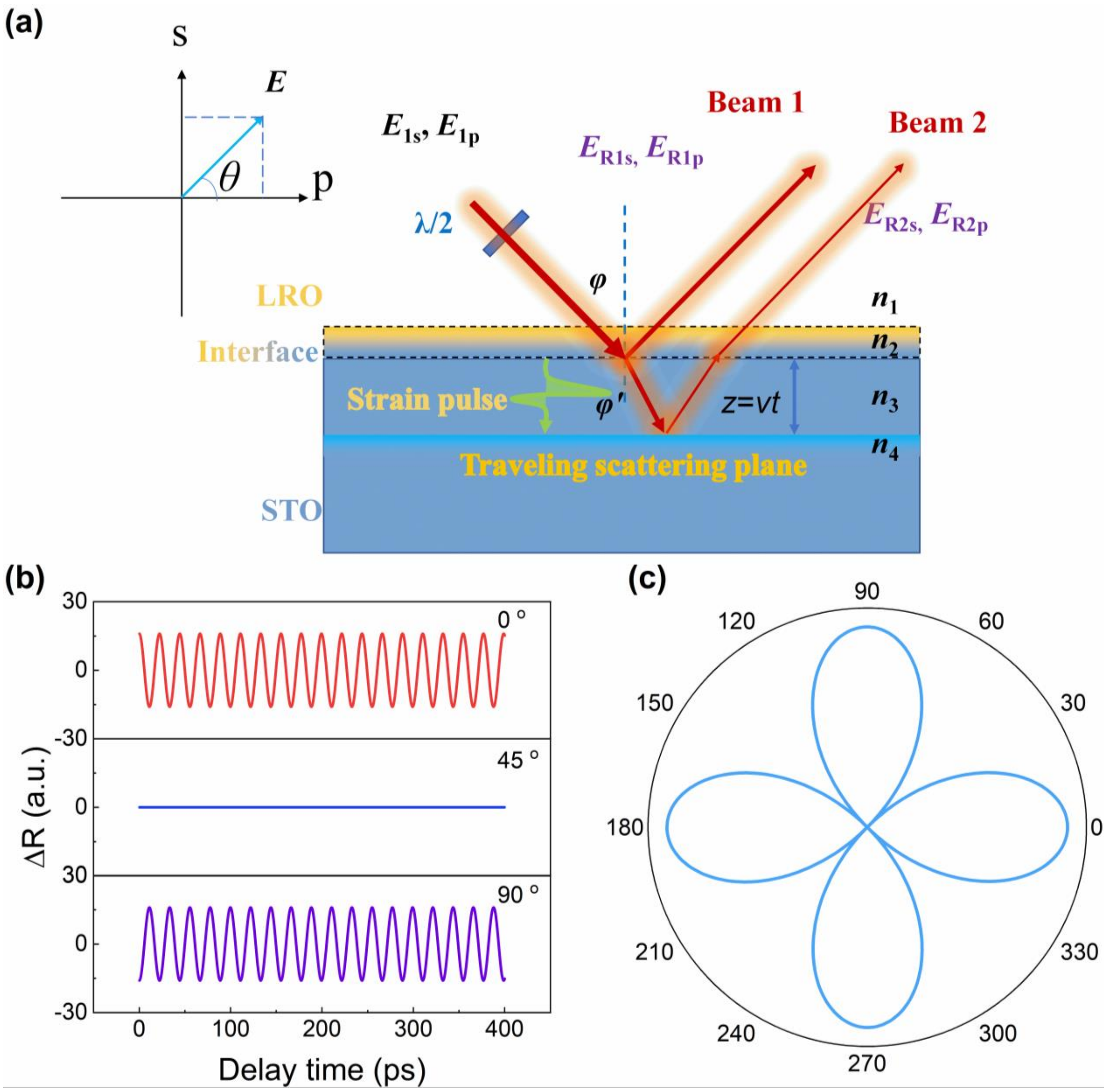

Figure S7. Simulated results of polarization dependence of transient reflectivity oscillations for the probe beam. (a) Schematic sketch of the experimental setup and the detection principle of the time-resolved all-optical pump-probe. (b) Simulated transient reflectivity oscillations (TR-ΔR) for probe polarization angles at 0° (p-polarization), 45°, and 90° (s-polarization). (c) Simulated amplitude of the transient reflectivity oscillations versus the polarization angles of the probe beam.

An ultrashort optical pump pulse is partially absorbed by a thin LRO transducer layer, and a strain pulse was generated by the instantaneous thermal expansion[1], which could be regarded as a wave packet arising from the superposition of longitudinal acoustic phonons having different wave vectors[2]. The acoustic pulse travels in the STO layer at the speed of sound *v* and induces a refractive

index modulation, which constitutes a traveling scattering plane inside STO crystal. The schematic sketch is shown in Figure S7(a), the input probe beam ($\boldsymbol{E}_1$) was injected into the LRO/STO heterostructure at an angle of φ to surface normal, and beam 1 and beam 2 were reflected from by the interface and the traveling scattering plane (at the position of $z = vt$), respectively.

To simulate the polarization dependence of the transient reflectivity oscillations, the interaction between the reflected beam 1 and beam 2 was considered in the numerical simulation. The laser intensity $I(P)$ detected by the photoelectric detector can be considered as follows:

$$\begin{aligned} I(P) &= I_{\mathrm{R1}}(P) + I_{\mathrm{R2}}(P) + 2\sqrt{I_{\mathrm{R1}}(P)\, I_{\mathrm{R2}}(P)}\cos\delta \\ &= E_{\mathrm{R1}}^2 + E_{\mathrm{R2}}^2 + 2E_{\mathrm{R1}}E_{\mathrm{R2}}\cos\delta \end{aligned} \quad \text{(S1)}$$

Here, δ is the phase difference between beam 1 and beam 2 at the photodetector, $\boldsymbol{E}_{\mathbf{R1}}$ and $\boldsymbol{E}_{\mathbf{R2}}$ are the complex electric field values for the reflected beam 1 and beam 2, respectively. According to the principle of vector decomposition, linearly polarized light can be decomposed into **s** and **p** polarized components. Linearly polarized light with the polarization angle θ can be considered as follows:

$$\vec{E} = E\cos(\theta)\,\vec{\mathrm{s}} + E\sin(\theta)\,\vec{\mathrm{p}} \quad \text{(S2)}$$

Therefore, equation (1) can be written as:

$$\begin{aligned} I(P) &= E_{\mathrm{R1}}^2 + E_{\mathrm{R2}}^2 + 2E_{\mathrm{R1}}E_{\mathrm{R2}}\cos\delta \\ &= \left(E_{\mathrm{R1s}}^2 + E_{\mathrm{R2s}}^2 + 2E_{\mathrm{R1s}}E_{\mathrm{R2s}}\cos\delta_{\mathrm{s}}\right) + \left(E_{\mathrm{R1p}}^2 + E_{\mathrm{R2p}}^2 + 2E_{\mathrm{R1p}}E_{\mathrm{R2p}}\cos\delta_{\mathrm{p}}\right) \\ &= \left(E_{\mathrm{R1}}^2 + E_{\mathrm{R2}}^2 + 2E_{\mathrm{R1}}E_{\mathrm{R2}}\cos\delta_{\mathrm{s}}\right)\cos^2(\theta) + \left(E_{\mathrm{R1}}^2 + E_{\mathrm{R2}}^2 + 2E_{\mathrm{R1}}E_{\mathrm{R2}}\cos\delta_{\mathrm{p}}\right)\sin^2(\theta) \\ &= I(P_{\mathrm{s}})\cos^2(\theta) + I(P_{\mathrm{p}})\sin^2(\theta) \end{aligned} \quad \text{(S3)}$$

Here, δ_s and δ_p are phase differences between beam 1 and beam 2 are for the **s** and **p** polarized components at the photodetectors, respectively. Based on Fermat's principle, δ_s and δ_p can be written as:

$$\delta_p = 2\pi\left(2v_s t\sqrt{n^2 - \sin^2(\varphi)}\right)\Big/\lambda_{probe}$$
$$\delta_s = \pi + \left[2\pi\left(2v_s t\sqrt{n^2 - \sin^2(\varphi)}\right)\Big/\lambda_{probe}\right] \quad \text{(S4)}$$

Here, n = 2.34 is the refractive index of STO at 800 nm[3], v_s stands for the value of longitudinal sound velocity (8088 m s^{-1}), λ_{probe} represents the probe wavelength in vacuum, t is the delay time and φ is the probe incidence angle (45°). For 110 oriented STO, there is a difference of π between δ_s and δ_p due to the half-wave loss of **s** component,

Based on this simple model, the TR-ΔR at probe polarization angels of 0° (**p**-polarization), 45°, and 90° (**s**-polarization) were numerically simulated, as shown in Figure S7(b). From the simulated results, the amplitudes of the transient reflectivity oscillations are large at 0° and 90°, while the amplitude at 45° is hard to be observed, which are similar to the experimental results in Figure 3(c). The amplitude of the transient reflectivity oscillations versus the polarization angle of the probe beam was also numerically simulated, as shown in Figure S7(c). The polarization dependence trace has four-fold symmetry with largest values at 0°, 90°, 180° and 270°, consistent with that observed in experiment as shown in Figure 3(b). From the above results, we can conclude that this simple mode can explain the oscillation behavior in the ultrafast reflectivity changes.

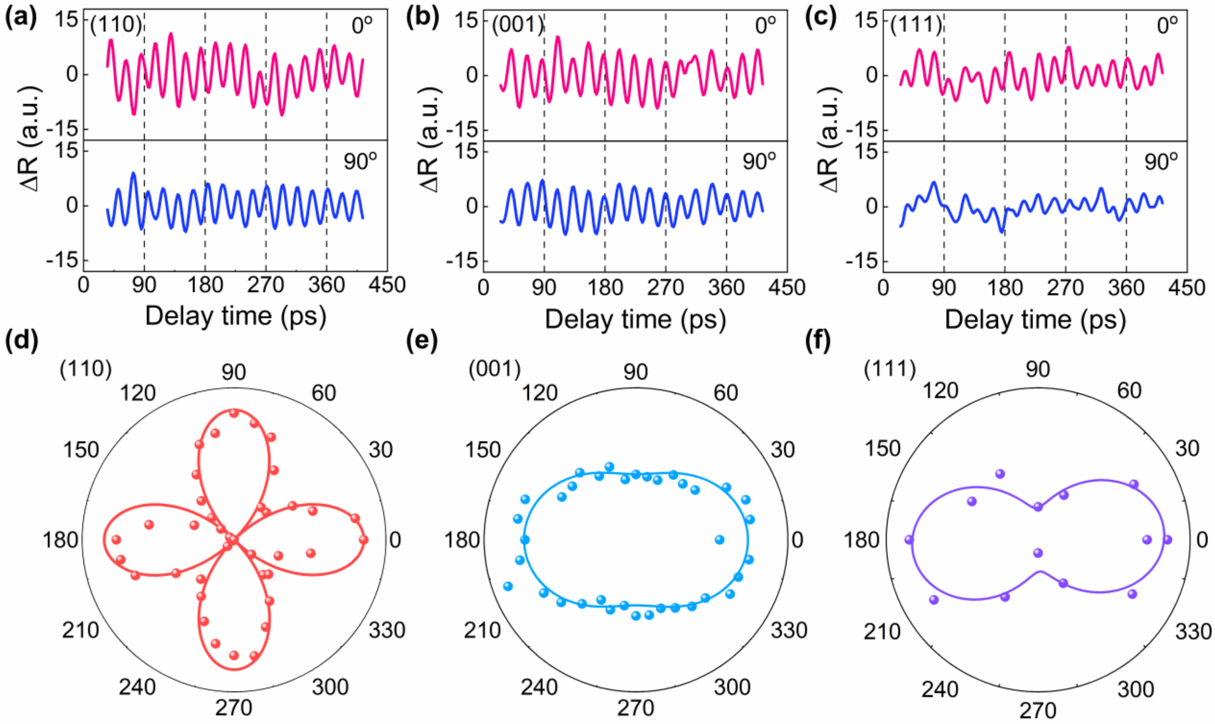

Figure S8. Polarization dependence of transient reflectivity oscillation at different lattice orientations. (a), (b), and (c) Transient reflectivity oscillation spectra of LRO/STO (110), LRO/STO (111), and LRO/STO (001) at probe polarization angels of 0° (p-polarization) and 90° (s-polarization). (d), (e), and (f) Amplitude of TR-ΔR versus polarization angles. The solid curves denote the fitted data.

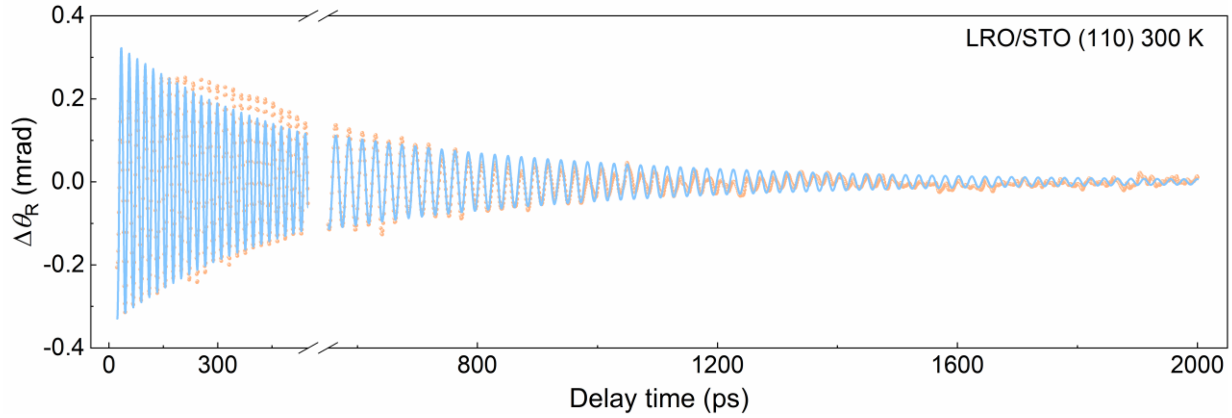

Figure S9. The optical birefringence oscillation $\Delta\theta_R$ of the LRO(68 nm)/STO (110) structure measured at 300 K.

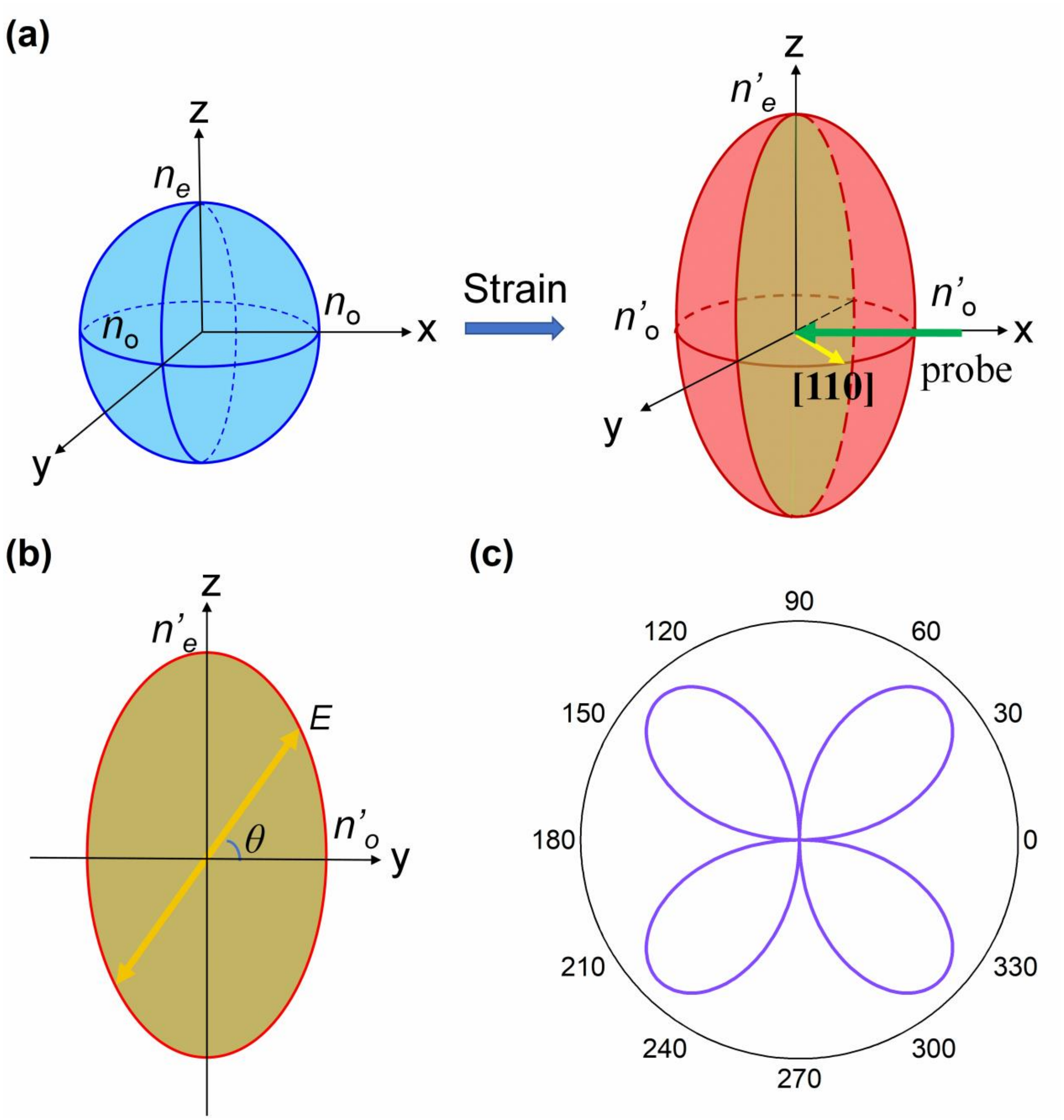

Figure S10. Schematic sketch of refractive index ellipsoid stretching of (110) orientation induced by the strain and simulated amplitude of the optical birefringence $\Delta\theta_R$ versus probe polarization angle. (a) The schematic sketch of refractive index ellipsoid stretching of STO (110). (b) The refractive index elliptic plane perpendicular to the incident probe beam. The orange arrow represent the polarization direction of probe beam. (c) The simulated amplitude of the optical birefringence $\Delta\theta_R$ versus probe polarization angle.

The longitudinal strain pulse will induce the transient change on the principal diagonal elements of the dielectric tensor of STO, resulting in changes of the refractive index ellipsoid, stretching or compression. For the STO with (110) orientation, the angle between the incident direction of the

probe beam and the surface normal of the sample is 45°, so the transmission direction of the incident probe beam is [100]. Therefore, the sectional projection of the refractive index ellipsoid in the YZ plane needs to be considered. The angle between the polarization direction of the probe beam and the Y-axis ([010]) is θ, and the Jones vector of the probe laser is $\tilde{E}_0 = e^{i(kz-\omega t)}\begin{bmatrix} E_{0x}e^{i\varphi_x} \\ E_{0y}e^{i\varphi_y} \end{bmatrix} = A\cdot\begin{bmatrix} \cos\theta \\ \sin\theta \end{bmatrix}$.

After the strain pulse excitation, the STO Jones matrix is $\begin{bmatrix} 1 & 0 \\ 0 & e^{i\Gamma} \end{bmatrix}$.

Where the Γ represents the phase difference between the ordinary (**o**) or extraordinary (**e**) components for the probe light at the STO strain pulse interface.

$$\Gamma \sim \frac{2\pi\left(n_e^{'} - n_o^{'}\right)}{\lambda_{probe}}$$

After the reflection at the STO strain pulse interface, the Jones vector for the probe laser can be expressed as:

$$\begin{bmatrix} 1 & 0 \\ 0 & e^{i\Gamma} \end{bmatrix}\cdot\begin{bmatrix} \cos\theta \\ \sin\theta \end{bmatrix} = \begin{bmatrix} \cos\theta \\ \sin\theta e^{i\Gamma} \end{bmatrix} = \begin{bmatrix} \cos\theta \\ \sin\theta\left(\cos\Gamma + i\sin\Gamma\right) \end{bmatrix} = \begin{bmatrix} \cos\theta \\ \sin\theta\cos\Gamma + i\sin\theta\sin\Gamma \end{bmatrix}$$

At this point, the polarization angle of the probe laser is:

$$\alpha = \arctan\left(\tan\theta\left(\cos\Gamma + i\sin\Gamma\right)\right) \approx \arctan\left(\tan\theta\cos\Gamma\right)$$

Since the change of polarization angle is only within the magnitude of mrad range, the change of polarization angle of the probe laser is:

$$\Delta\theta = \arctan(\tan\theta\cos\Gamma) - \theta .$$

Based on this simple model, the amplitude of the probe polarization rotation angle versus probe polarization angle was numerically simulated, as shown in Figure S10, which holds a quadruple symmetry, and the maximum values are obtained when the polarization angles are 45°, 135°, 225°, and 315°, similar to the experimental results in Figure 3(e). Therefore, this simple model can explain

the amplitude dependence of the rotation angle of the probe beam.

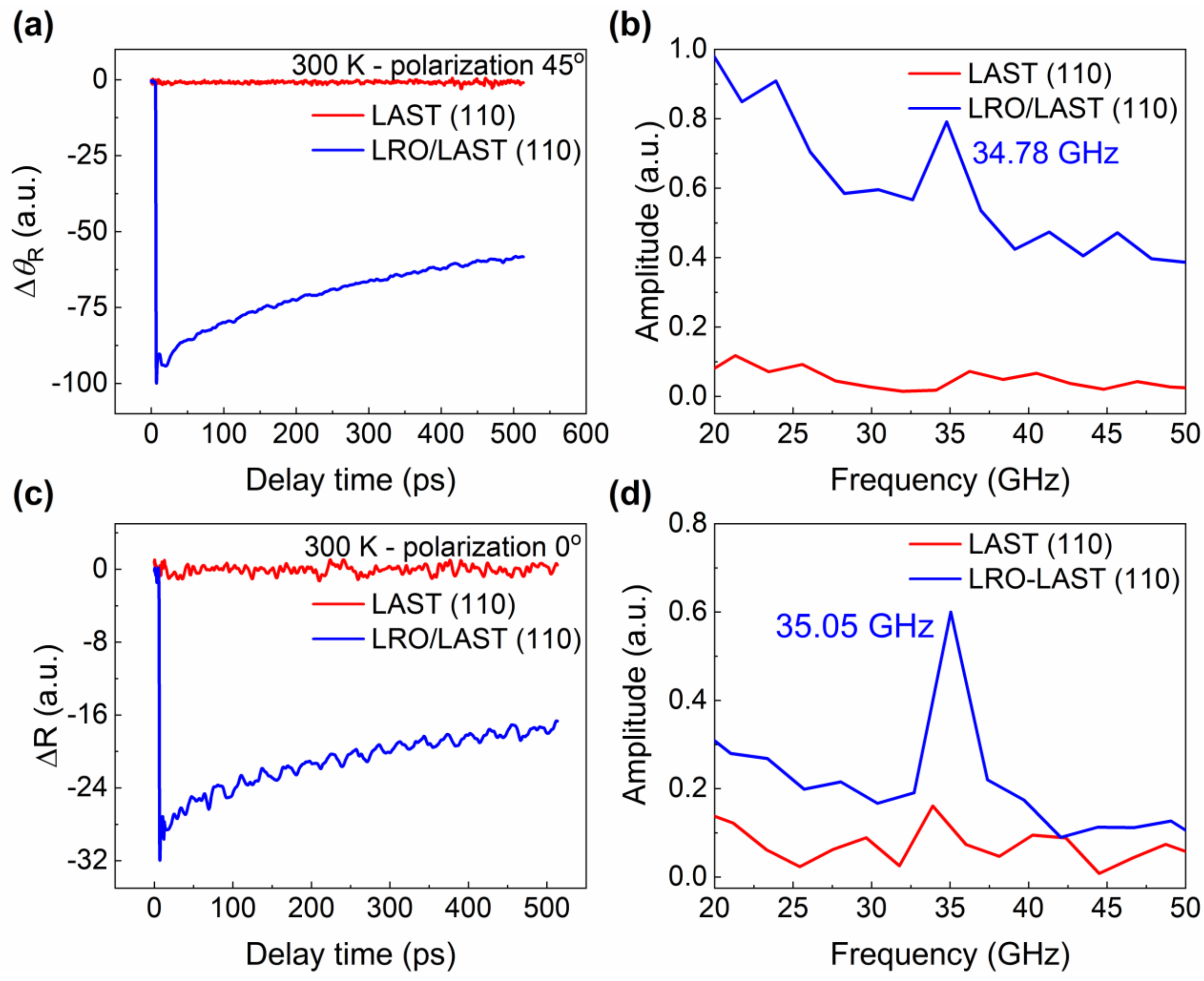

Figure S11. The optical birefringence and transient reflectivity data of the LAST (110) substrate and LRO/LAST (110) structure. (a), (c) Polarization rotation angle and reflectivity spectra versus the scanning delay time for these two samples. (b), (d) The corresponding Fourier transform data for the data in the Figure S11(a), (c) and the center frequencies are similar (~35 GHz) , which are different from the results of STO-based samples (~45 GHz).

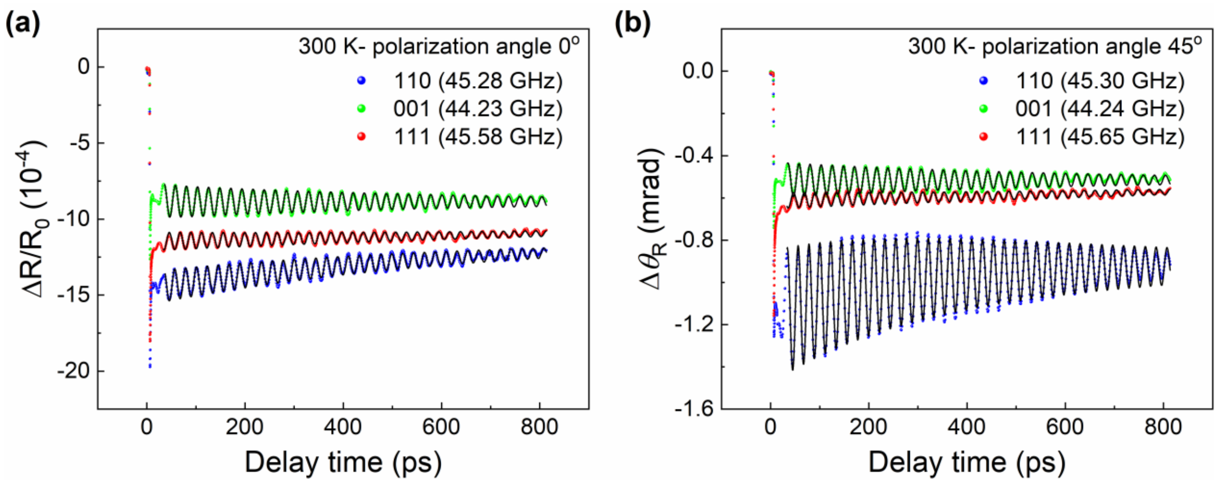

Figure S12. The optical birefringence and transient reflectivity data from different lattice orientations of STO. (a), (b) Transient reflectivity spectra $\Delta R/R_0$ (probe polarization angle 0°) and the probe polarization rotation angle $\Delta\theta_R$ (probe polarization angle 45°) for LRO/STO at different lattice orientations of STO. The solid curves denote the fitted data.

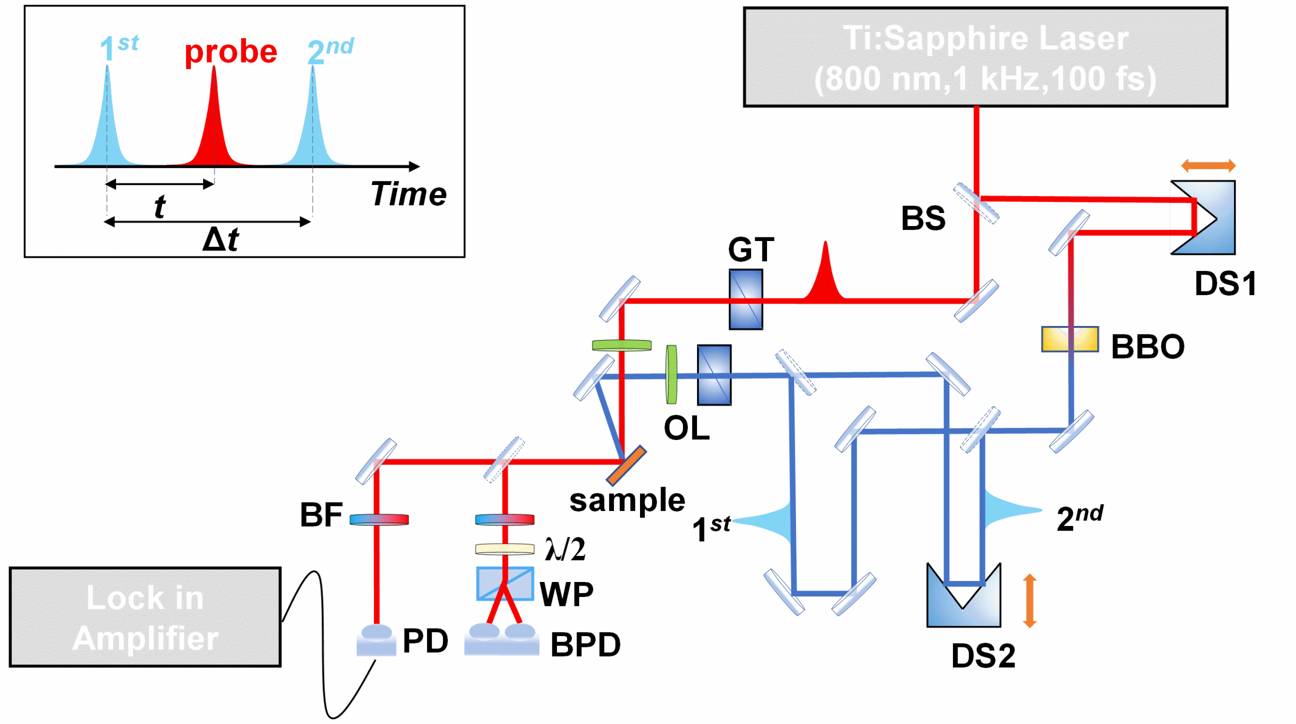

Figure S13. The schematic view of the double pump technique measurement system. The components are as follows: a barium borate crystal (BBO), a Glan-Thompson polarizer (GT), an optical lens (OL), a beam splitter (BS), a photoelectric detector (PD), a balance photoelectric detector (BPD), bandpass filter (BF), a Wollaston prism (WP), a half-wave plate (λ/2), and two delay stages (DS1 and DS2). Inset: Timing chart of the probe and the two pump pulses.

To realize the coherent phonons and optical birefringence manipulation in the LRO/STO structure, time-resolved optical reflectivity (TR-ΔR) measurement with two pump pulses was performed. The experimental setup and detection principle were shown in the schematic in Figure S13. Sample were kept at room temperature. The light source was a Ti:sapphire pulse laser with a wavelength of 800 nm, a repetition rate of 1 kHz, and a pulse duration of 100 fs. A beam splitter was used to divide the laser output into two parts. After converting the pump pulse wavelength to 400 nm with a barium borate crystal (BBO) by second harmonic generation (SHG) process, the pump beam was further divided into two optical beams by another beam splitter. To control the time delay between the pump pulses of the two beams (Δ*t*), a delay stage (DS2) was placed in the one of pump beams. A positive

sign of Δt means that the pump pulse of the beam that passed through DS2 (the "second" pump pulse) arrived later at the sample surface than the pump pulse of the other beam (the "first" pump pulse). The relative delay (t) between the first pump pulse and the probe pulse is scanned by the DS1 delay stage. The incident angle of the probe beam is ~45° with respect to the normal direction of the sample plane, while the pump was ~35° to the surface normal. The beams sizes are the same as the single pump experiment as mentioned above. Both pump 1 and pump 2 fluences are 9.6 mJ cm^{-2}. The pump beam was chopped at a rate of 635 Hz to measure the relative changes in the reflectance between the pump perturbed ($R_0 + \Delta R$) and unperturbed (R_0) samples. A low noise photodetector (New Focus, Model 2007) and a lock-in amplifier (Zurich Instruments, MFLI 500 kHz) are used to improve the signal-to-noise ratio. To track the transient optical birefringence effect $\Delta\theta_R$, the reflected light from the sample was first filtered to remove the pump, passed through a half-wave plate and a Wollaston prism, and then detected by a pair of balanced photodiodes. The pump-induced change in the rotation of the polarization angle was determined as the ratio of the intensity imbalance/the sum intensity of each photodiode obtained from a lock-in amplifier locked at the pump modulation frequency.

References

[1] C. Thomsen, H. T. Grahn, H. J. Maris, J. Tauc, *Phys. Rev. B* **1986**, 34, 4129.
[2] S. Brivio, D. Polli, A. Crespi, R. Osellame, G. Cerullo, R. Bertacco, *Appl. Phys. Lett.* **2011**, 98, 211907
[3] M. J. Weber, *Handbook of optical materials*, CRC Press, Boca Raton, FL, USA **2018**.
